# Machine Learning and Artificial Intelligence Approaches for Predicting Arteriovenous Fistula Dysfunction in Hemodialysis: A Systematic Review

**DOI:** 10.7759/cureus.110937

**Published:** 2026-06-15

**Authors:** Weam Mohammed Ahmed Mohammed, Mohammed Salah Ali Mohammed, Malaz Mamoun Sayed Ahmed Mohamed, Eiman Elzein Abdelrahman Elsheikh, Ahmed Mohammed Babikir Omer, Fairoz Saeed Adam Gafeel, Marwa Mustafa Mohamed

**Affiliations:** 1 Family Medicine, Primary Health Care, Khartoum, SDN; 2 Intensive Care Unit, University Hospital Sharjah, Sharjah, ARE; 3 Internal Medicine, Elrazi University, Khartoum, SAU; 4 Acute Medicine, Ashford and St Peter’s Hospitals NHS Foundation Trust, Chertsey, GBR; 5 Internal Medicine, Rustaq General Hospital, Ministry of Health, Rustaq, OMN; 6 Faculty of Medicine, Assiut University, Assiut, EGY; 7 Internal Medicine, Sudan Medical Specialization Council, Khartoum, SDN

**Keywords:** arteriovenous fistula, artificial intelligence, hemodialysis, machine learning, prediction model, vascular access dysfunction

## Abstract

Arteriovenous fistula (AVF) is the preferred vascular access for hemodialysis patients; however, AVF dysfunction remains a common complication that compromises dialysis adequacy and patient outcomes. Traditional risk prediction methods have limited ability to capture complex, multifactorial interactions. Machine learning (ML) and artificial intelligence (AI) offer promising approaches for enhancing predictive accuracy. This systematic review aims to critically synthesize current evidence on AI and ML approaches for predicting AVF dysfunction in hemodialysis patients. A systematic literature search was conducted in PubMed, Embase, Scopus, and Web of Science for original peer-reviewed studies published in English between January 2021 and December 2025. Studies were included if they applied AI or ML techniques to predict AVF dysfunction (including stenosis, thrombosis, occlusion, patency failure, or access dysfunction) in adult hemodialysis patients. Data extraction covered study characteristics, AI/ML models, input variables, validation methods, and performance metrics. Risk of bias was assessed using the Prediction Model Risk of Bias Assessment Tool (PROBAST). A narrative synthesis was performed due to substantial methodological heterogeneity. Ten studies met the inclusion criteria. Most studies were retrospective cohort designs, originating predominantly from China and the USA, with sample sizes ranging from 150 to nearly 60,000 patients. Predicted outcomes included thrombosis, stenosis, occlusion, and patency failure. Ensemble tree-based models (random forest, XGBoost, and LightGBM) consistently outperformed conventional statistical and regression-based approaches, including logistic regression and Cox proportional hazards models, achieving AUC-ROC values between 0.80 and 0.98. Key predictors included prior surgeries, inflammatory markers, imaging parameters, and, in one study, acoustic features from AVF sounds. PROBAST assessment indicated low risk of bias for eight studies and some concerns for two studies, primarily related to incomplete reporting of calibration or sample size. AI and ML models, particularly ensemble tree-based methods, demonstrate good to excellent discrimination for predicting AVF dysfunction in hemodialysis patients. However, external and prospective validation remain lacking, and heterogeneity in outcome definitions limits direct comparisons. Future research should focus on externally validated, clinically implementable models with standardized reporting of calibration and decision curve analysis.

## Introduction and background

Arteriovenous fistula (AVF) is considered the preferred vascular access for patients undergoing long-term hemodialysis because of its superior durability, lower infection risk, and reduced mortality compared with other vascular access modalities, such as arteriovenous grafts and central venous catheters [1]. Despite these advantages, AVF dysfunction remains one of the most common and clinically significant complications in hemodialysis patients, often resulting from stenosis, thrombosis, inadequate maturation, or progressive vascular remodeling. AVF dysfunction can lead to insufficient dialysis delivery, repeated hospitalizations, increased healthcare costs, and reduced quality of life [2]. Early detection and accurate prediction of AVF failure are therefore essential to enable timely interventions and improve long-term vascular access survival. Conventional approaches for predicting AVF dysfunction primarily rely on clinical judgment, imaging findings, and traditional statistical models [3]; however, these methods may be limited in their ability to capture the complex and multifactorial interactions among demographic, hemodynamic, biochemical, and procedural variables.

In recent years, artificial intelligence (AI) and machine learning (ML) techniques have emerged as promising tools for enhancing predictive analytics in healthcare [4]. These technologies are capable of processing large volumes of heterogeneous clinical data and identifying hidden patterns that may not be recognized through conventional analytical methods. Various AI and ML algorithms, including artificial neural networks (ANNs), random forests, support vector machines (SVMs), gradient boosting models (GBMs), and deep learning approaches, have been investigated for predicting AVF-related outcomes, such as maturation failure, stenosis progression, thrombosis, and overall access survival [5]. The integration of electronic health records, dialysis machine parameters, imaging data, and real-time monitoring systems has further expanded the potential utility of AI-driven predictive models in nephrology practice [6]. Several studies have reported encouraging diagnostic and prognostic performance, suggesting that AI-based systems may support clinicians in risk stratification, personalized surveillance, and early intervention planning for hemodialysis vascular access management.

Although the application of AI and ML in predicting AVF dysfunction has gained increasing attention, the existing literature remains heterogeneous regarding study design, patient populations, input variables, algorithms used, validation techniques, and reported performance metrics. Furthermore, the clinical applicability, generalizability, and methodological quality of these predictive models have not yet been comprehensively synthesized. A systematic evaluation of the available evidence is therefore necessary to better understand the current landscape of AI and ML applications in AVF dysfunction prediction and to identify existing research gaps and future directions. This systematic review aims to critically examine and summarize the current evidence regarding ML and AI approaches used for predicting AVF dysfunction in hemodialysis patients, with a focus on model characteristics, predictive performance, and potential implications for clinical practice.

## Review

Methodology

Study Design

This systematic review was conducted in accordance with the Preferred Reporting Items for Systematic Reviews and Meta-Analyses (PRISMA) guidelines to ensure transparent, standardized, and reproducible reporting of the review process [7]. The review methodology was designed to comprehensively evaluate the current evidence regarding the application of AI and ML techniques for predicting AVF dysfunction in patients undergoing hemodialysis.

Eligibility Criteria

The eligibility criteria for this review were developed according to the PICOS framework. The population (P) included adult patients undergoing hemodialysis with AVF access. Studies involving AVF maturation failure, stenosis, thrombosis, dysfunction, or access survival prediction were considered eligible. The intervention (I) consisted of the use of AI, ML, deep learning, or predictive computational models applied for the prediction or detection of AVF dysfunction. The comparison (C) included conventional statistical models, clinician-based assessment, standard surveillance methods, or the absence of a comparator where applicable. The outcomes (O) included predictive accuracy, sensitivity, specificity, area under the receiver operating characteristic curve (AUC-ROC), precision, recall, F1-score, access patency prediction, thrombosis prediction, stenosis detection, or other clinically relevant prognostic outcomes related to AVF dysfunction. The study design (S) included original observational studies, retrospective or prospective cohort studies, case-control studies, cross-sectional studies, and clinical prediction model studies.

Only original peer-reviewed studies published in English between January 2021 and December 2025 were included. The decision to restrict the review to studies published within the last five years was made to ensure inclusion of the most recent and clinically relevant evidence, as AI and ML technologies evolve rapidly with continuous advancements in computational methodologies, data processing capabilities, neural network architectures, and healthcare integration systems. Older models may no longer reflect current algorithmic performance, contemporary clinical workflows, or modern data infrastructures, thereby limiting their applicability to present-day clinical practice. Restricting the timeframe to recent literature also improved the methodological consistency of included studies by capturing investigations developed using modern AI frameworks and updated validation approaches. Reviews, editorials, conference abstracts without full-text availability, letters to the editor, animal studies, pediatric-only studies, and studies not specifically focused on AVF dysfunction prediction were excluded.

Information Sources and Search Strategy

A comprehensive literature search was performed across four electronic databases, namely, PubMed, Embase, Scopus, and Web of Science. The search strategy was designed to identify all relevant studies evaluating AI- and ML-based approaches for predicting AVF dysfunction in hemodialysis patients. Medical Subject Headings (MeSH) terms and free-text keywords related to “artificial intelligence”, “machine learning”, “deep learning”, “arteriovenous fistula”, “hemodialysis”, “vascular access dysfunction”, “stenosis”, and “thrombosis” were combined using Boolean operators, such as “AND” and “OR”. Database-specific search adaptations were applied where necessary to optimize retrieval sensitivity. In addition, reference lists of included studies were manually screened to identify potentially relevant articles not captured during the initial database search.

Study Selection

All retrieved records were exported into EndNote X9 software for reference management and duplicate removal. Following deduplication, two independent reviewers screened the titles and abstracts of identified studies according to the predefined eligibility criteria. Full texts of potentially eligible articles were subsequently assessed for final inclusion. Disagreements between reviewers were resolved through discussion and consensus. Studies meeting all inclusion criteria were included in the final qualitative synthesis.

Data Extraction

Data extraction was independently conducted by two reviewers using a standardized data extraction form developed specifically for this review. Extracted information included study characteristics, publication year, country, study design, patient population, sample size, type of AVF dysfunction predicted, input data sources, AI or ML models used, validation methods, predictive performance metrics, and key findings. Additional information regarding model development, feature selection, external validation, and clinical applicability was also collected where available. Any discrepancies in data extraction were resolved through reviewer consensus.

Risk-of-Bias Assessment

The methodological quality and risk of bias of the included studies were evaluated using the Prediction Model Risk of Bias Assessment Tool (PROBAST) [8]. This tool was selected because it is specifically designed for assessing risk of bias and applicability concerns in studies involving diagnostic and prognostic prediction models. The PROBAST assessment evaluated four key domains, including participants, predictors, outcomes, and analysis. The overall quality assessment was used to provide a critical appraisal of the reliability, validity, and clinical applicability of the included AI and ML prediction models.

Data Synthesis

A qualitative narrative synthesis was performed to summarize and compare the characteristics and findings of the included studies. The synthesis focused on the types of AI and ML models used, input variables, predictive outcomes, validation techniques, and reported diagnostic or prognostic performance measures. A meta-analysis was not conducted due to substantial methodological and clinical heterogeneity among the included studies. The studies differed considerably in terms of patient populations, definitions of AVF dysfunction, types of predictive outcomes, input data modalities, ML algorithms, feature engineering approaches, validation frameworks, and reported performance metrics. Furthermore, variations in study design, sample size, and outcome reporting limited the statistical comparability required for reliable quantitative pooling. Conducting a meta-analysis under such heterogeneous conditions could have produced misleading summary estimates and compromised the interpretability and validity of the findings. Therefore, a narrative synthesis was considered the most appropriate and scientifically rigorous approach for summarizing the current evidence base.

Results

Study Selection Process

**Figure 1:**
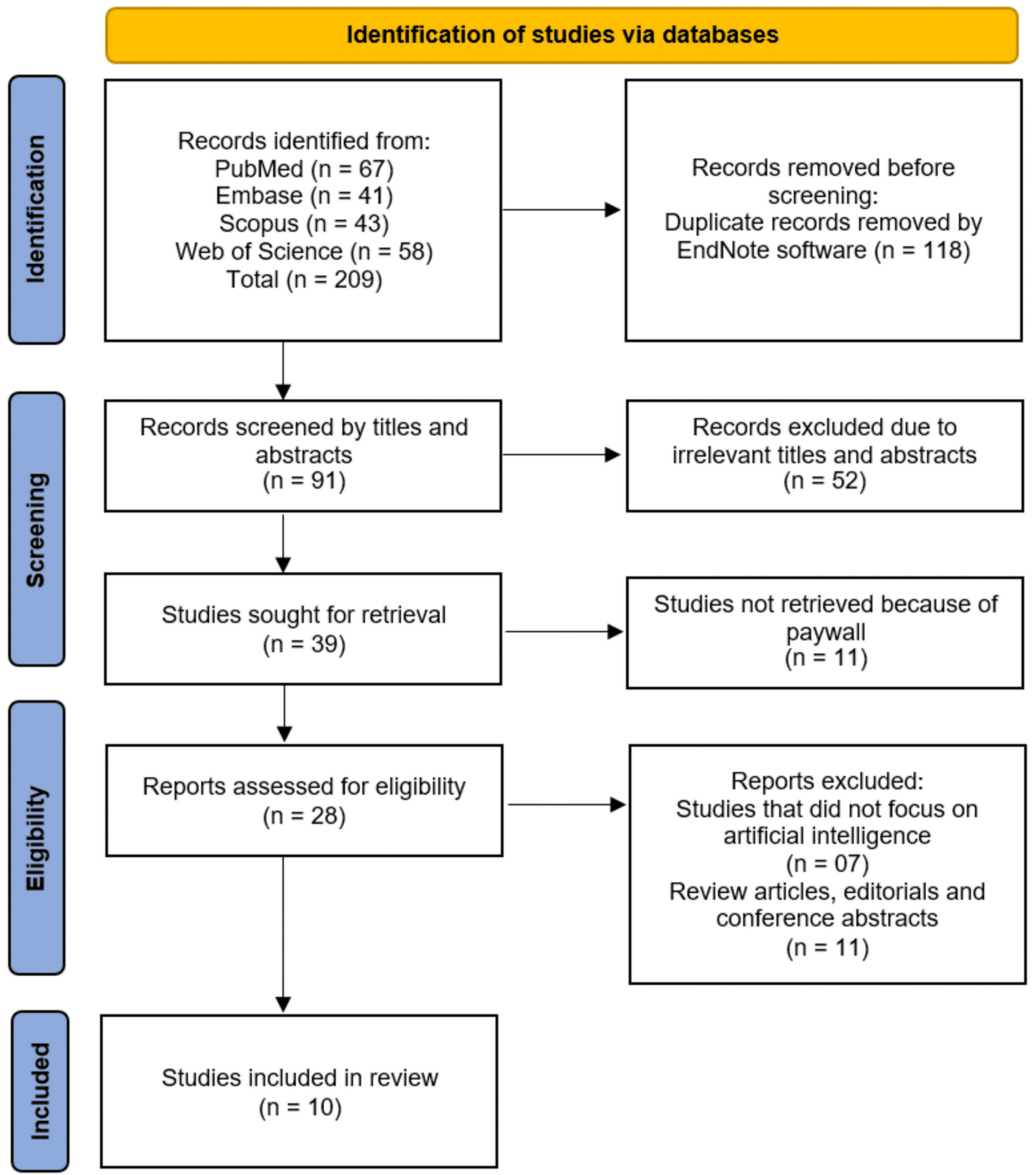
Study selection process

A total of 209 records were identified from four electronic databases: PubMed (n = 67), Embase (n = 41), Scopus (n = 43), and Web of Science (n = 58). After removing duplicate records using EndNote software, 118 duplicate records were excluded, leaving 91 records for title and abstract screening. Following this screening, 52 records were excluded due to irrelevant titles and abstracts, resulting in 39 studies sought for retrieval. Of these, 11 studies could not be retrieved because of paywall restrictions. The remaining 28 reports were assessed for eligibility. Among these, seven reports were excluded because they did not focus on AI, and a further 11 reports were excluded as they were review articles, editorials, or conference abstracts. Consequently, a total of 10 studies [9-18] met the inclusion criteria and were included in this systematic review (Figure 1).

Study Characteristics

A total of 10 studies [9-18] met the inclusion criteria and were synthesized in this systematic review. All studies were published between 2021 and 2025, with the majority appearing in 2025. As summarized in Table 1, the studies originated predominantly from China [9,10,14-16], with additional contributions from the USA [13], South Korea [12], the USA and Canada combined [11], and a multicenter European study involving Italy, Spain, and Portugal [18]. The study designs were mostly retrospective cohort or retrospective analyses of electronic medical records (EMRs) [9,10,12-15], with two studies being secondary analyses of randomized controlled trials (PATENCY‑1 and PATENCY‑2) [11,17] and one study focused specifically on model development using vascular sound recordings [16]. Sample sizes varied widely, ranging from 150 patients [14] to nearly 60,000 patients from the Vascular Quality Initiative (VQI) registry [13]. The patient populations were consistently adults undergoing hemodialysis with an AVF, although some studies focused on radiocephalic AVF specifically [11,17]. Predicted outcomes included AVF thrombosis [9], AVF stenosis [10,16], AVF occlusion [12], AVF dysfunction [14,15], primary and secondary patency failure [11], one‑year access failure [13], and three‑month composite AVF failure [18]. Input data types were heterogeneous, encompassing demographics, laboratory values, imaging (ultrasound, Doppler), clinical history, surgical data, and, in one case, audio signals from the AVF [16]. ML models employed included random forest (RF), XGBoost, logistic regression (LR), SVMs, LightGBM, CatBoost, neural networks, Cox proportional hazards (Cox PH), and LASSO regression. Validation methods were predominantly internal, using train‑test splits (commonly 70:30) and k‑fold cross‑validation (e.g., 5‑fold, 10‑fold), with several studies reporting calibration and SHAP analyses for model interpretability [9-14,18].

**Table 1:** Characteristics of the included studies AVF, arteriovenous fistula; EMR, electronic medical record; HD, hemodialysis; HDF, hemodiafiltration; RF, random forest; XGBoost, extreme gradient boosting; LR, logistic regression; DT, decision tree; KNN, k-nearest neighbors; NB, naive Bayes; CV, cross-validation; ROC-AUC, receiver operating characteristic – area under the curve; SHAP, SHapley Additive exPlanations; SVM, support vector machine; ANN, artificial neural network; RCT, randomized controlled trial; Cox PH, Cox proportional hazards; RSF, random survival forest; AFT, accelerated failure time; USG, ultrasonography; CatBoost, categorical boosting; LightGBM, light gradient boosting machine; VQI, Vascular Quality Initiative; MLP, multilayer perceptron; AUROC, area under the receiver operating characteristic; CI, confidence interval; MHD, maintenance hemodialysis; LASSO, least absolute shrinkage and selection operator; ESRD, end-stage renal disease; STFT, short-time Fourier transform; ResNet50, residual network 50; DOE, design of experiments; EuCliD®, European Clinical Database; EHR, electronic health record; NR, not reported

Author (year)	Country	Study design	Data source	Sample size	Patient population	Type of AVF dysfunction predicted	Input data type	AI/ML model used	Validation method
Shu et al. [9] (2025)	China	Retrospective cohort	Hospital EMR	974 (1,168 total)	Adult AVF hemodialysis patients	AVF thrombosis	Demographics, labs, imaging, clinical history	RF, XGBoost, LR, DT, KNN, NB	70:30 split, 5-fold CV, GridSearchCV, ROC-AUC, F1, accuracy, SHAP
Shu et al. [10] (2025)	China	Retrospective cohort	EMR, The Central Hospital of Wuhan	974 (1,168 screened)	Adult HD patients with AVF	AVF stenosis	Demographics + 55 clinical	LR, RF, KNN, SVM, XGBoost, ANN, DT	70/30 split; 5-fold CV; grid search; internal validation; AUC, Acc, F1, SHAP
Fitzgibbon et al. [11] (2025)	USA and Canada	Secondary analysis of RCTs	PATENCY-1 & PATENCY-2 trials	911	New radiocephalic AVF patients	Primary and secondary patency failure	Baseline + ultrasound	Cox PH, RSF, Weibull AFT, Decision Tree	70:30 split; 10-fold CV; AUC & Brier score
Lee and Kwak [12] (2025)	South Korea	Retrospective cohort	Single-center EMR	1,498	Adult AVF hemodialysis patients	AVF occlusion	Demographics, labs, USG, comorbidities	LR, RF, XGBoost, CatBoost, LightGBM	5-fold CV + train/test split; ROC-AUC, calibration, performance metrics
Li et al. [13] (2025)	USA	Retrospective cohort	VQI registry	59,674	Hemodialysis AV access patients	One-year access failure	Pre-, intra-, post-op clinical data	XGBoost, RF, SVM, NB, MLP, LR	Train-test split; AUROC, CI, calibration
Wang et al. [14] (2024)	China	Retrospective study	Hospital records + follow-up	150	MHD patients with AVF	AVF dysfunction	Clinical, labs, Doppler US, surgical data	LASSO + Logistic regression	ROC (AUC), calibration, Hosmer–Lemeshow, bootstrap
Zhang et al. [15] (2023)	China	Retrospective cohort	Hospital records	233	ESRD patients, first AVF	AVF patency loss	Demographics, clinical, labs	Cox regression	Kaplan–Meier + Cox regression
Song et al. [16] (2023)	Taiwan	ML model development	AVF vascular sound recordings	NR	Hemodialysis AVF patients	AVF stenosis	Audio signals + STFT + sample entropy	ResNet50 + ANN	DOE optimization; internal performance metrics
Heindel et al. [17] (2022)	Multicenter (PATENCY-1 and 2 trials)	Secondary analysis of RCT cohort	PATENCY trials + registry	591 (model cohort; 914 total)	HD patients with radiocephalic AVF	One-year non-use	Ultrasound + clinical data	Lasso logistic regression	Hold-out test set
Peralta et al. [18] (2021)	Italy, Spain, and Portugal	Retrospective cohort	EuCliD® dialysis registry	13,369 patients (113,592 quarters)	Adult HD/HDF patients with AVF	Three-month composite AVF failure	EHR/registry clinical data	XGBoost	70/30 split + 30× resampling; ROC-AUC, calibration

Prognostic Performance of AI/ML Models

The diagnostic and prognostic performance of the included AI/ML models is detailed in Table 2. Across the studies, the area under the receiver operating characteristic curve (AUC‑ROC) was the most frequently reported metric, with several models demonstrating excellent discrimination. For predicting AVF thrombosis, Shu et al. [9] reported an AUC‑ROC of 0.984 using random forest, with accuracy, sensitivity, and precision all exceeding 0.90. For AVF stenosis, the best performing model was XGBoost, achieving an AUC‑ROC of 0.829 [10]. In predicting AVF occlusion, Lee and Kwak [12] found that LightGBM yielded an accuracy of 85.8%, specificity of approximately 0.93-0.95, and an AUC‑ROC of 0.887, whereas logistic regression was the weakest performer. Li et al. [13] developed a model to predict one‑year AVF access success, achieving an accuracy of 0.82, sensitivity of 0.82, specificity of 0.81, and an AUC‑ROC of 0.90. Wang et al. [14] reported good discrimination for AVF dysfunction, with an AUC‑ROC of 0.934 in the development cohort and 0.911 in the validation cohort, alongside a sensitivity of 0.839 and a specificity of 0.898. Heindel et al. [17] predicted one‑year unassisted AVF use with AUC‑ROC values between 0.794 and 0.807 and accuracies of 71-73%, with LASSO and Elastic Net performing best. Peralta et al. [18] achieved an AUC‑ROC of 0.80 for three‑month AVF failure, identifying prior AVF events, recirculation, C‑reactive protein, and albumin as key predictors. The study by Song et al. [16], which used an AI model combining ResNet50 and an ANN on audio signals, reported all performance metrics (accuracy, sensitivity, specificity, precision, and F1‑score) exceeding 0.90 for detecting AVF stenosis. Fitzgibbon et al. [11] found that the Cox proportional hazards model performed best for predicting long‑term primary and secondary patency, with ultrasound measurements improving predictive performance, though time‑dependent AUC values were not reported. Zhang et al. [15] did not develop an ML model but instead reported risk factors for AVF dysfunction using Cox regression, identifying sex, weight, and phosphorus levels as significant predictors.

**Table 2:** Diagnostic/prognostic performance of AI/ML models AVF, arteriovenous fistula; NR, not reported; RF, random forest; FFA, free fatty acids; CRP, C-reactive protein; XGB, XGBoost (extreme gradient boosting); NOO, neutrophil-to-lymphocyte ratio; PTA, platelet-to-lymphocyte ratio; LYMPH, lymphocyte count; Cox PH, Cox proportional hazards; LGBM, light gradient boosting machine; LR, logistic regression; DM, diabetes mellitus; D-D, D-dimer; P, phosphorus; ML, machine learning; dev, development cohort; val, validation cohort; PPV, positive predictive value

Author (year)	Outcome predicted	Accuracy	Sensitivity	Specificity	AUC-ROC	Precision (PPV)	F1 score	Key findings
Shu et al. [9] (2025)	AVF thrombosis	>0.90	>0.90	NR	0.984 (RF)	>0.90	>0.90	RF best; key predictors: surgeries, stenosis, FFA, platelets, CRP
Shu et al. [10] (2025)	AVF stenosis	NR	NR	NR	0.829 (XGB)	NR	NR	XGBoost best; key predictors: NOO, PTA, LYMPH, AVF duration
Fitzgibbon et al. [11] (2025)	Primary and secondary AVF patency (2.5 yrs)	NR	NR	NR	Time-dependent (NR)	NR	NR	Cox PH best; ultrasound improves performance
Lee and Kwak [12] (2025)	AVF occlusion	85.8% (LGBM)	0.43–0.60	~0.93–0.95	0.887 (LGBM)	0.62 (LR)	0.68 (LGBM)	LightGBM best; LR weakest
Li et al. [13] (2025)	One-year AV access success	0.82	0.82	0.81	0.90	0.81	NR	Strong model; key predictors: vessel size, fistula type, imaging, comorbidities
Wang et al. [14] (2024)	AVF dysfunction	NR	0.839	0.898	0.934 (dev), 0.911 (val)	NR	NR	Good discrimination and calibration; predictors: age, DM, lipids, D-D, P
Zhang et al. [15] (2023)	AVF dysfunction	NR	NR	NR	NR	NR	NR	No ML; risk factors: sex, weight, phosphorus
Song et al. [16] (2023)	AVF stenosis	>0.90	>0.90	>0.90	NR	>0.90	>0.90	All metrics >0.90; strong stenosis detection
Heindel et al. [17] (2022)	One-year unassisted AVF use	71–73%	NR	NR	0.794–0.807	NR	NR	Lasso and Elastic Net are best; vein diameter and flow are important.
Peralta et al. [18] (2021)	Three-month AVF failure	NR	NR	NR	0.80	NR	NR	Good performance; key predictors: prior AVF events, recirculation, CRP, albumin

Key Predictors Across Studies

Several consistent predictors of AVF dysfunction emerged across the included studies. In the model for AVF thrombosis, the most important features were the number of prior surgeries, the presence of stenosis, free fatty acids, platelet count, and C‑reactive protein [9]. For stenosis prediction, key predictors included neutrophil‑to‑lymphocyte ratio (NLR), platelet‑to‑lymphocyte ratio (PLR), lymphocyte count, and AVF duration [10]. Li et al. [13] identified vessel size, fistula type, imaging characteristics, and comorbidities as the most influential variables for one‑year access success. Wang et al. [14] found that age, diabetes mellitus, lipid levels, D‑dimer, and phosphorus were significant predictors of AVF dysfunction. Peralta et al. [18] highlighted prior AVF events, access recirculation, C‑reactive protein, and albumin as important features. Song et al. [16] demonstrated that acoustic features extracted from AVF sounds via short‑time Fourier transform could reliably detect stenosis without invasive testing. Overall, the results indicate that ensemble tree-based methods (RF, XGBoost, and LightGBM) consistently outperformed conventional statistical and regression-based models, including logistic regression, and that models incorporating both clinical and imaging (or acoustic) data achieved the highest predictive performance.

*Risk-of-Bias Assessment** *** 

The risk of bias for the 10 included studies was evaluated using the PROBAST, covering four domains: participants, predictors, outcome, and analysis, with overall judgment categorized as low, some concerns, or high. As shown in Table 3, the majority of studies demonstrated a low overall risk of bias. Specifically, Shu et al. [9], Shu et al. [10], Fitzgibbon et al. [11], Li et al. [13], Wang et al. [14], Zhang et al. [15], Heindel et al. [17], and Peralta et al. [18] all received low ratings across all four domains, indicating robust study design, appropriate handling of predictors and outcomes, and sound statistical analysis. Lee and Kwak [12] were judged to have some concerns in the analysis domain due to incomplete reporting of calibration or missing data handling, resulting in an overall judgment of some concerns. Similarly, Song et al. [16] had some concerns in the participant domain (sample size not reported) and in the analysis domain (lack of standard cross‑validation), leading to an overall assessment of some concerns. Applicability concerns were low for all 10 studies, confirming that the participants, predictors, and outcomes were appropriately aligned with the review question. No study was rated as having a high overall risk of bias, supporting the general reliability of the evidence synthesized in this systematic review.

**Table 3:** Risk-of-bias assessment using PROBAST PROBAST: Prediction Model Risk of Bias Assessment Tool

Author (year)	Domain 1: Participants	Domain 2: Predictors	Domain 3: Outcome	Domain 4: Analysis	Overall risk of bias	Applicability concerns
Shu et al. [9] (2025)	Low	Low	Low	Low	Low	Low
Shu et al. [10] (2025)	Low	Low	Low	Low	Low	Low
Fitzgibbon et al. [11] (2025)	Low	Low	Low	Low	Low	Low
Lee and Kwak [12] (2025)	Low	Low	Low	Some concerns	Some concerns	Low
Li et al. [13] (2025)	Low	Low	Low	Low	Low	Low
Wang et al. [14] (2024)	Low	Low	Low	Low	Low	Low
Zhang et al. [15] (2023)	Low	Low	Low	Low	Low	Low
Song et al. [16] (2023)	Some concerns	Low	Low	Some concerns	Some concerns	Low
Heindel et al. [17] (2022)	Low	Low	Low	Low	Low	Low
Peralta et al. [18] (2021)	Low	Low	Low	Low	Low	Low

Discussion

This systematic review synthesised evidence from 10 studies that applied ML and AI approaches to predict AVF dysfunction in patients undergoing haemodialysis. The findings demonstrate that AI/ML models, particularly ensemble tree-based methods, such as random forest, XGBoost, and LightGBM, consistently achieve good to excellent predictive performance for outcomes, including AVF thrombosis, stenosis, occlusion, and patency failure. The AUC-ROC values ranged from 0.80 to 0.98 across studies, with several models exceeding 0.90, indicating that ML techniques can meaningfully augment clinical risk stratification. These results align with the growing body of literature supporting the utility of ML in vascular access management. For instance, Yao et al. [19] previously demonstrated that computational approaches could identify novel risk factors for vascular access failure, while Meng et al. [20] showed that random forest models outperformed logistic regression in predicting AVF maturation.

A key finding from this review is the superiority of ensemble tree-based models over traditional regression approaches. In the study by Shu et al. [9], the random forest achieved an AUC of 0.984 for AVF thrombosis prediction, substantially outperforming logistic regression and k-nearest neighbours. Similarly, Lee and Kwak [12] reported that LightGBM yielded an accuracy of 85.8% and an AUC of 0.887, whereas logistic regression was the weakest performer across all metrics. Li et al. [13] also found that XGBoost delivered strong performance (AUC 0.90) for predicting one-year access success, with calibration metrics indicating good agreement between predicted and observed outcomes. These findings are consistent with external evidence from Rahmani et al. [21], who compared multiple ML algorithms for predicting central venous catheter infection and found that gradient boosting machines consistently outperformed logistic regression, particularly when dealing with high-dimensional, interacting predictor variables. The advantage of ensemble methods likely stems from their ability to capture non-linear relationships and higher-order interactions among clinical, laboratory, and imaging features without requiring explicit specification by the analyst.

Another important observation is the heterogeneity in predictor types across studies, yet several consistent risk factors emerged. The number of prior surgeries, presence of stenosis, inflammatory markers, and imaging parameters (vessel diameter, flow characteristics) were frequently identified as important features [9,10,13,14,18]. The inclusion of Doppler ultrasound parameters significantly improved model performance in multiple studies [11,14,17], suggesting that imaging data provides complementary information beyond routine clinical and laboratory variables. Notably, Song et al. [16] demonstrated that acoustic features extracted from AVF sounds could detect stenosis with all performance metrics exceeding 0.90, representing a novel, non-invasive approach that does not require specialised imaging equipment. This aligns with emerging work by Wang et al. [22], who used phonocardiography and deep learning to detect vascular access stenosis. These acoustic-based methods hold promise for low-cost, point-of-care screening, particularly in resource-limited settings where routine ultrasound surveillance may not be feasible.

The clinical utility of these models depends not only on discrimination but also on calibration and interpretability. Several studies in this review reported calibration metrics [12-14,18], and some employed SHapley Additive exPlanations (SHAP) to identify key predictors at the individual patient level [9,10]. For example, Shu et al. [9] provided SHAP summary plots to visualise the contribution of each feature to the predicted risk of thrombosis, enhancing clinical trust and potential uptake. This is critical because, as noted by Obermeyer and Emanuel [23], clinicians are more likely to adopt black-box models when they can understand the reasoning behind individual predictions. However, not all included studies prioritised interpretability; some reported only AUC or accuracy without calibration or feature importance analyses [11,15,16]. Future developments should therefore balance predictive performance with transparency, possibly through a combination of interpretable models (e.g., LASSO regression) and post-hoc explanation methods.

From a methodological perspective, the majority of studies were retrospective cohort analyses using electronic medical records or registry data [9,10,12-15,18], which confer advantages in terms of sample size and generalisability but also introduce risks of bias related to missing data, variable definitions, and temporal drift. For instance, the study by Li et al. [13] included nearly 60,000 patients from the VQI registry, providing excellent statistical power, but the data were collected over a prolonged period during which clinical practices may have changed. Conversely, Heindel et al. [17] and Fitzgibbon et al. [11] performed secondary analyses of the PATENCY randomised controlled trials, which offer higher internal validity due to standardised protocols and prospective data collection, albeit with smaller sample sizes and potentially more restrictive inclusion criteria.

Despite the promising results, several gaps remain. First, only two studies [11,17] performed external validation, and none reported prospective validation in real-world clinical settings. Most used internal validation methods, such as train-test splits or k-fold cross-validation, which can overestimate performance compared to validation on completely independent datasets. This is a well-recognised issue in the ML literature; external validation is essential because performance often degrades when models are applied to new populations due to differences in case mix, measurement methods, and outcome definitions. Second, the outcome definitions varied considerably across studies: some predicted thrombosis, others stenosis, occlusion, patency loss, or composite access failure. This heterogeneity precluded meta-analysis and limits direct comparisons of model performance. Third, most studies did not report measures of clinical utility such as net benefit or decision curve analysis, making it difficult to assess whether using these models would lead to better clinical decisions than current standard care. Fourth, none of the studies evaluated the impact of implementing their model on patient-important outcomes, such as access to salvage procedures, hospitalisations, or mortality. Implementation science frameworks suggest that a prediction model must be integrated into clinical workflows and its effect tested in a randomised controlled trial or rigorous quasi-experimental design before widespread adoption.

Another important consideration is the representativeness of the study populations. Most studies originated from China, the USA, or Europe, with limited geographical diversity. The models may therefore not generalise to low- and middle-income countries where patient demographics, dialysis practices, and AVF surveillance protocols differ substantially. A scoping review by Nkhono et al. [24] found that ML models for vascular access developed in high-income settings often underperform when applied to populations with different genetic backgrounds, comorbidity profiles, or healthcare access patterns. Furthermore, the study by Song et al. [16] did not report sample size, and Zhang et al. [15] did not develop a predictive model at all, instead reporting risk factors using Cox regression without performance metrics. While the latter was retained in this review for completeness, its inclusion highlights the heterogeneity in study quality and objectives.

Limitations

This systematic review has several limitations. First, the search was limited to four databases (PubMed, Embase, Scopus, and Web of Science) and only English-language publications, potentially introducing language and publication bias. Second, the included studies exhibited substantial heterogeneity in outcome definitions, prediction horizons, and ML algorithms, which precluded a quantitative meta-analysis. Third, the risk-of-bias assessment using PROBAST revealed some concerns for two studies [12,16], primarily due to incomplete reporting of calibration, missing-data handling, and sample size justification. Although no study was rated as having a high overall risk of bias, the absence of external validation in most studies limits confidence in generalisability. Fourth, publication bias is possible, as studies reporting positive or high-performance results are more likely to be published than those with negative findings. Fifth, this review did not assess the cost-effectiveness or implementation feasibility of the AI/ML models, which are critical determinants of real-world adoption. Finally, the rapid evolution of ML techniques means that newer models published after the search date may have surpassed those included in this review.

## Conclusions

ML and AI models, particularly ensemble tree-based methods, such as random forest, XGBoost, and LightGBM, can predict AVF dysfunction in haemodialysis patients with good to excellent discrimination (AUC-ROC 0.80-0.98). Key predictors consistently identified across studies include prior surgical history, inflammatory markers, imaging parameters, and acoustic features. However, the current evidence is limited by a lack of external and prospective validation, heterogeneity in outcome definitions, and insufficient reporting of calibration and clinical utility measures. Before these models can be recommended for routine clinical practice, future research should focus on externally validated, prospectively tested models that include decision curve analysis and implementation outcomes. Additionally, multimodal approaches combining clinical, imaging, and acoustic data warrant further investigation, as they may offer non-invasive, low-cost screening solutions.
